# A genome-wide screen for human salicylic acid (SA)-binding proteins reveals targets through which SA may influence development of various diseases

**DOI:** 10.1038/s41598-019-49234-6

**Published:** 2019-09-11

**Authors:** Hyong Woo Choi, Lei Wang, Adrian F. Powell, Susan R. Strickler, Dekai Wang, D’Maris A. Dempsey, Frank C. Schroeder, Daniel F. Klessig

**Affiliations:** 1 Boyce Thompson Institute, Ithaca, New York 14853 USA; 2 Department of Plant Medicals, Andong National University, Andong, 36729 Korea; 3 Present Address: College of life sciences and medicine, Zhejiang Sci-Tech University, Hangzhou, 310018 China

**Keywords:** Proteins, Molecular medicine

## Abstract

Salicylic acid (SA) is the major metabolite and active ingredient of aspirin; both compounds reduce pain, fever, and inflammation. Despite over a century of research, aspirin/SA’s mechanism(s) of action is still only partially understood. Here we report the results of a genome-wide, high-throughput screen to identify potential SA-binding proteins (SABPs) in human HEK293 cells. Following photo-affinity crosslinking to 4-azidoSA and immuno-selection with an anti-SA antibody, approximately 2,000 proteins were identified. Among these, 95 were enriched more than 10-fold. Pathway enrichment analysis with these 95 candidate SABPs (cSABPs) revealed possible involvement of SA in multiple biological pathways, including (i) glycolysis, (ii) cytoskeletal assembly and/or signaling, and (iii) NF-κB-mediated immune signaling. The two most enriched cSABPs, which corresponded to the glycolytic enzymes alpha-enolase (ENO1) and pyruvate kinase isozyme M2 (PKM2), were assessed for their ability to bind SA and SA’s more potent derivative amorfrutin B1 (amoB1). SA and amoB1 bound recombinant ENO1 and PKM2 at low millimolar and micromolar concentrations, respectively, and inhibited their enzymatic activities *in vitro*. Consistent with these results, low millimolar concentrations of SA suppressed glycolytic activity in HEK293 cells. To provide insights into how SA might affect various human diseases, a cSABP-human disorder/disease network map was also generated.

## Introduction

For millennia, plants containing high levels of SA and its derivatives (collectively termed salicylates) have been used therapeutically by cultures throughout the world. Even today, these plants are used as alternatives to non-steroidal anti-inflammatory drugs (NSAIDs). In the fourth century B.C., Hippocrates reportedly prescribed extracts of willow leaves or bark to treat fever and to provide pain relief during childbirth^1^. Willow leaves and bark are rich in the salicylate salicin, which is converted to SA upon ingestion. During the middle of the nineteenth century, medicinal use of SA increased drastically. To satisfy the heightened demand, commercial production of synthetic SA was begun in 1874. After 1897, acetylated SA (aspirin) replaced the use of SA because it had fewer negative side effects, such as stomach irritation and bleeding^2^. In addition to relieving pain, fever, and inflammation, aspirin has been shown to reduce the risk of stroke, heart attack, and some cancers when taken prophylactically. The demonstration that salicylate-based drugs exert beneficial effects on a number of chronic and devastating diseases, such as type II diabetes, Alzheimer’s disease and certain types of cancers, suggests that future studies will identify an even more expansive role for salicylates as therapeutic agents^3–8^.

Efforts to understand aspirin’s mechanism of action led to the discovery that aspirin suppresses prostaglandin synthesis by irreversibly inhibiting the cyclooxygenases COX1 and COX2^9^. Prostaglandins are hormone-like compounds that cause pain, fever, and inflammation. This important discovery, made in the 1970s, provided the basis for the biomedical community’s prevailing view that aspirin primarily works by inhibiting these two enzymes. However, this hypothesis fails to explain how SA can elicit nearly the same pharmacological effects as aspirin even though SA is a poor inhibitor of these cyclooxygenases. Furthermore, aspirin is metabolized to SA within minutes in the human body, while SA is stable for hours. These facts, combined with the observations that (i) SA was the primary drug used to reduce pain, fever, and inflammation before aspirin was developed, and (ii) salicylate-rich medicinal plants have and continue to be used extensively worldwide, argue that there must be additional targets through which SA/aspirin exert their many pharmacological effects.

Fifteen potential targets of SA and/or SA prodrugs, such as aspirin, salsalate and sulfasalazine, have been identified over the past 30 years^2^. Several of these are associated with inflammation, including high mobility group box 1 (HMGB1), tumor necrosis factor alpha (TNFα), nuclear factor-kappa B (NF-κB), and inhibitor of NF-κB kinase subunit beta (IKK-β). At low millimolar concentrations, SA and aspirin have been shown to suppress the activation of NF-κB, a key regulator of genes associated with immune and inflammatory responses^9^. NF-κB is sequestered in the cytoplasm by inhibitor of NF-κB (IκB); it is released after the IκB kinase (IKK) complex phosphorylates IκB, which promotes IκB degradation^10,11^. SA/aspirin were found to suppress NF-κB activation by inhibiting IκB degradation^9^; however, whether they directly bind and inhibit the IKK-β subunit of IKK is controversial^10,11^.

Other proteins that have been identified as potential targets of SA include adenosine monophosphate-activated protein kinase (AMPK), a central regulator of cell growth and metabolism^12,13^. Millimolar concentrations of SA allosterically activated AMPK, which phosphorylates and thereby activates several metabolic enzymes^12,13^. By contrast, millimolar concentrations of SA inhibited the acetyltransferase activities of CREB-binding protein (CBP) and E1A binding protein P300 (P300) by directly competing with acetyl-coenzyme A^7^. This inhibition correlated with reduced acetylation of (i) several histone proteins^7^, (ii) Alzheimer’s disease-associated tau^6^, and (iii) NF-κB^7^, which requires acetylation for full activation. At low micromolar concentrations, SA was reported to inhibit the pro-inflammatory activities of HMGB1 and the cell death-mediating activity of glyceraldehyde 3-phosphate dehydrogenase (GAPDH)^14,15^. Strikingly, natural and synthetic derivatives of SA that display an even greater ability to inhibit these two targets have been identified.

To better understand how SA and its derivatives exert their beneficial, as well as adverse, effects we undertook a genome-wide search for human SABPs. This screen was based on an approach employed previously to identify 29 new SABPs and several dozen cSABPs in plants, where SA is an endogenous hormone that regulates multiple processes, including immunity (http://bioinfo.bti.cornell.edu/SA2010/). SA’s small size (138.12 Da) makes identifying SABPs particularly challenging, as it limits the number of contacts SA can make within its binding site in an SABP. This, in turn, often leads to weak and/or transient SA-SABP interactions. Additionally, SA’s size facilitates weak, non-specific interactions with pseudo-sites on non-target proteins, which leads to many false positives. To overcome these problems, we used the photo-reactive SA derivative 4-azidoSA (4AzSA). Upon UV irradiation, the photo-reactive azido group (R‒N = N^+^ = N^‒^) forms a nitrene group (R‒N:) that can covalently bond with certain C-H and N-H sites in the amino acid side chains or peptide backbone of an interacting protein. The 4AzSA-crosslinked proteins were then immuno-selected with a very high specificity anti-SA antibody and identified by mass spectrometry^16,17^. This screen identified approximately 2,000 proteins, including some previously reported SA target proteins. To reduce the chance of selecting proteins that non-specifically interact with 4AzSA, we performed additional enrichment analyses of these proteins against the published quantitative proteomic database^18^. This identified 95 highly enriched proteins, which we termed cSABPs. Two of the most enriched cSABPs were shown to bind SA, and their enzyme activities were inhibited by SA and its natural derivative amoB1.

## Results

### Genome-wide screening for SA target proteins

**Figure 1:**
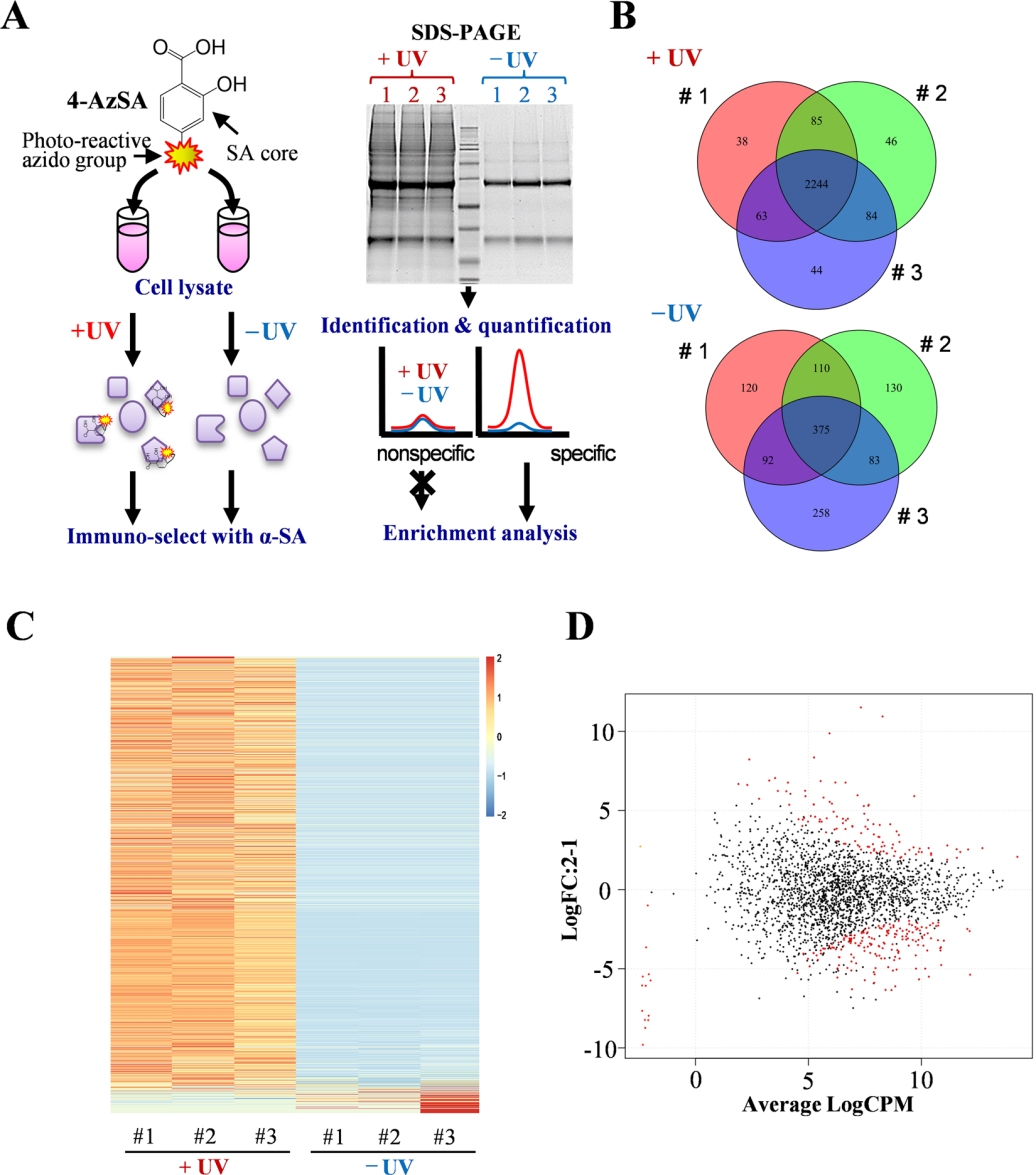
Genome-wide screening for human SA-binding proteins (SABPs). (**A**) Schematic depiction of screening for SABPs in HEK293 cell lysates using the photo-reactive SA analog 4AzSA (see Materials and Methods for more details). (**B**) Venn Diagram summarizing the overlap among proteins identified by the 4AzSA screening approach in three replicate samples (#1, #2 and #3) that were either subjected to UV crosslinking (+UV) or not (−UV). A total of 2,604 proteins were identified in the three samples exposed to UV irradiation, with 2,244 proteins found in all three replicates. In the absence of UV exposure, a total of 1168 proteins were identified, with 375 found in all three replicates. (**C**) Heat map of protein abundance in the three replicates (#1, #2 and #3) performed with (3 left lanes) or without (3 right lanes) UV exposure (+UV and −UV). (**D**) EdgeR scatter plot showing the quantitative enrichment of 2,329 4AzSA crosslinked, immuno-selected proteins as compared to their levels in the published HEK392 quantitative proteomic database. Differentially enriched proteins (with false discovery rate (FDR) < 0.05 and enrichment ratio >5) are shown in red.

To identify potential targets of SA in the human genome, we adapted a strategy previously used to identify SABPs in plants^16,17^. For this approach, three lysates of human embryonic kidney 293 (HEK293) cells were incubated with 4AzSA; half of each lysate was then subjected to photo-activated crosslinking by UV irradiation, whereas the other half, comprising a negative control, was not (Fig. 1A**)**. Proteins bound to 4AzSA were immuno-selected with an α-SA antibody coupled to protein G agarose resin, eluted from the resin with SA and fractionated by SDS–PAGE (Fig. 1A; Supplementary Fig. S1). Analysis of the SDS-PAGE gels revealed that the protein bands in the UV-irradiated samples (+UV) were both more numerous and more intense than those in the −UV control samples.

To facilitate the identification and quantification of potential SABPs, the SDS-PAGE gel was cut into four pieces and each section was analyzed by nano–HPLC–MS/MS (Supplementary Fig. S1). Section D, which corresponded to a ~50 kDa band and a ~25 kDa band present in all samples, contained the heavy and light chains of the IgG antibodies, respectively (Supplementary Fig. S1). For sections A, B, and C, 2,623 proteins were identified from both +UV and –UV samples (Supplementary Table S1**)**. In the +UV samples, 2,604 proteins with varying abundance were identified, of which 2,244 were found in all three replicates (Fig. 1B; Supplementary Table S2). In contrast, 1,168 proteins were identified in the −UV samples, of which 375 were found in all three replicates.

To increase the selection rigor, proteins that were present only in the +UV samples or had at least 10-fold greater average iBAQ values for the +UV samples than the −UV samples were identified. 2,329 of the 2,604 proteins met this criterion (Fig. 1C). A second selection criterion assessed whether the potential SABPs were quantitatively enriched in the screen as compared to their levels in the quantitative proteomic database of HEK293 cells^18^. One hundred fifty-nine proteins were significantly enriched, with a false discovery rate (FDR) < 0.05. Of these, 95 proteins satisfied the first selection criterion and also were enriched over 10-fold (Log FC > 3.3; FDR < 0.05) (Fig. 1D; Table 1; Supplementary Table S3); these proteins were designated cSABPs.

**Table 1:** List of 20 most enriched candidate SA-binding proteins.

Accession	LogFC	FDR^a^	Mass (kDa)	Name	MF^b^	Pathway
P06733	11.5	9.74E-34	47.2	ENO1	C^c^	Glycolysis
P14618	10.9	6.06E-34	57.9	PKM2	C	Glycolysis
P12814	9.9	3.13E-20	103.1	ACTN1		Integrin signaling
O75369	8.3	2.07E-20	278.2	FLNB		Integrin signaling
Q07954	8.2	7.76E-06	504.6	LRP1		Alzheimer disease-presenilin
Q96EP0	7.0	2.32E-07	119.7	HOIP/RNF31		NF-kB activation
Q13596	6.9	3.34E-07	59.1	SNX1	B^d^	
Q16891	6.9	4.07E-17	83.7	MIC60		
Q12906	6.8	3.85E-14	95.3	ILF3	B	
P17066	6.8	2.04E-21	71.0	HSPA6	B/C	Apoptosis signaling Parkinson's disease
Q9BQS8	6.8	4.46E-07	167.0	FYCO1		
Q86V85	6.7	8.53E-04	49.4	GPR180		insoluble
Q8TDY2	6.6	4.08E-04	183.1	RB1CC1	B/S^e^	insoluble
Q9NVH2	6.2	1.16E-08	106.8	INTS7		
P57088	6.2	1.03E-06	28.0	TMEM33	S	
Q13371	6.2	5.62E-06	34.3	PDCL		
P43243	6.1	9.21E-07	94.6	MATR3		
Q9Y6A5	6.0	2.72E-12	90.4	TACC3		
P61978	5.9	8.39E-18	51.0	HNRNPK	B/C	
Q04637	5.9	2.37E-08	175.5	EIF4G1	T^f^	

^a^false discovery rate, ^b^molecular function, ^c^catalytic activity, ^d^binding, ^e^structural molecule, ^f^translation regulator. See supplementary Table S3 for full list.

Analysis of these cSABPs revealed that they have wide range of functions. The glycolytic enzymes ENO1 and PKM2 had the highest enrichment scores, with a LogFC of 11.5 (~3,000-fold) and 10.9 (~2,000-fold), respectively (Table 1). Among the 28 proteins involved in the first two major stages of cellular respiration (glycolysis and the tricarboxylic acid [TCA] cycle), only ENO1 and PKM2 were highly enriched (Supplementary Fig. S2). Actin (ACTN1) and actin-binding protein filamin B (FLNB) were enriched ~1,000-fold and ~300-fold, respectively (Table 1). ACTN1 and FLNB both are cytoskeletal proteins involved in cell–cell contacts, cell–substratum adhesion, cell membrane remodeling, and cell motility^19^. The matri-cellular receptor low density lipoprotein (LDL) receptor-related protein 1 (LRP1) was enriched ~300-fold. LRP1 plays various roles in diverse biological processes, including lipoprotein metabolism, degradation of proteases, activation of lysosomal enzymes, and cellular entry of bacterial toxins and viruses^20^, as well as in several inflammation-associated diseases such as atherosclerosis, certain cancers, and injury to the nervous system^21^. HOIL-1-Interacting Protein (HOIP) was enriched ~100-fold. HOIP is an E3 component of the linear ubiquitin chain assembly complex (LUBAC) that regulates NF-κB activation via linear ubiquitination of NF-κB essential modulator (NEMO)^22^. The results of these analyses suggest that SABPs are involved in multiple pathways and/or cellular processes, including (i) glycolysis, (ii) cytoskeletal assembly and/or signaling, and (iii) NF-κB-mediated immune signaling.

### Reactome pathway analysis of cSABPs

**Figure 2:**
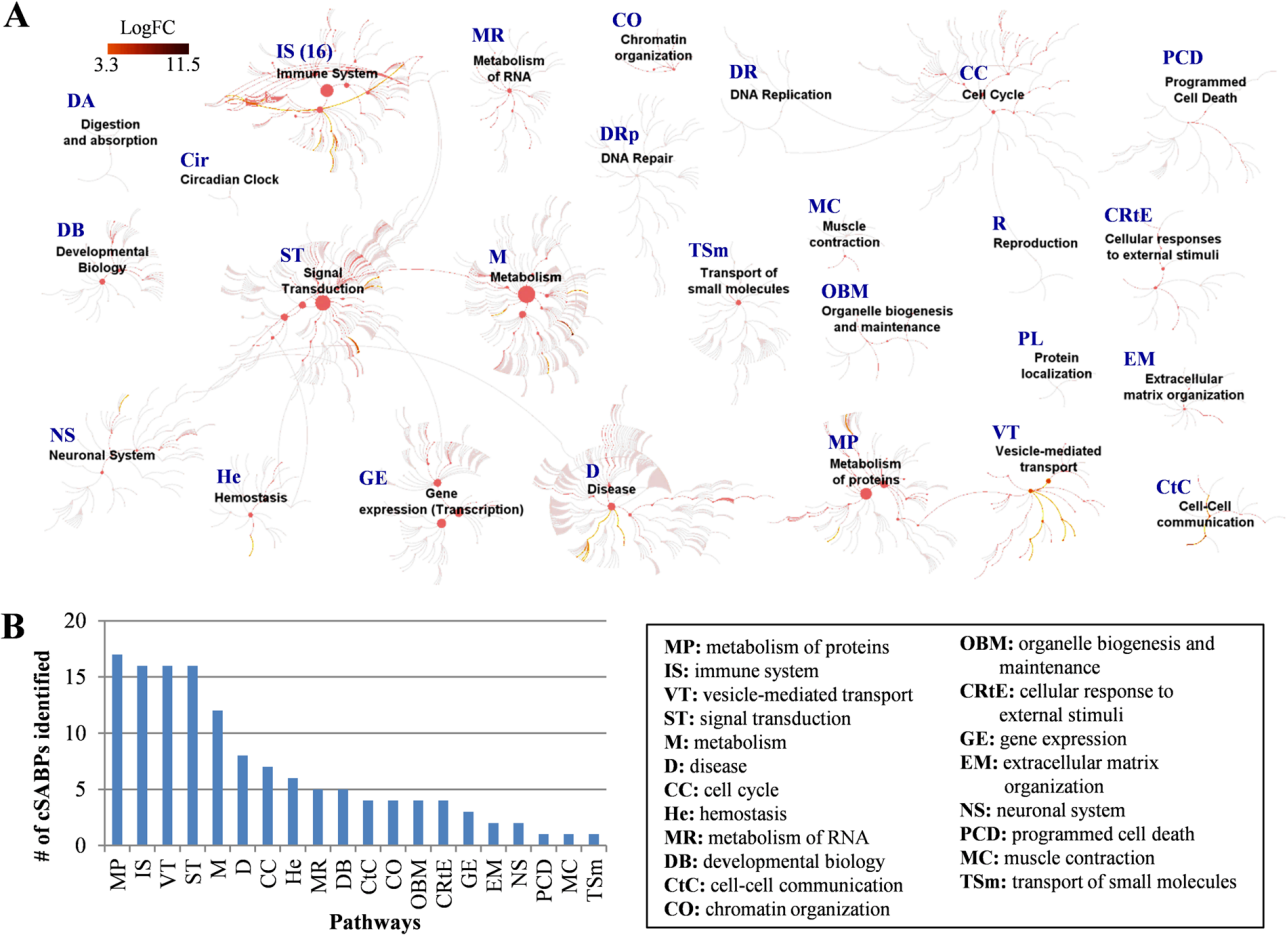
Pathway enrichment analysis of 95 cSABPs using the Reactome database (https://reactome.org/). (**A**) Biological pathways associated with the 95 cSABPs are highlighted by dark to bright red colors based on their enrichment levels (logFC) compared to their basal levels in the HEK 293 cells^18^. Note that a few reactome pathways, including digestion and ingestion (DA), circadian clock (Cir), DNA repair (Drp), DNA replication (DR) and reproduction (R), were not associated with any cSABPs. (**B**) Number of cSABPs found to be functional in different pathways. Explanation of pathway abbreviations is provided on the right.

To further investigate the cellular processes that may be affected by SA binding to cSABPs, pathway enrichment analysis was performed with the 95 cSABPs using the Reactome database (https://reactome.org/)^23^. This analysis revealed five pathways that each contain over 10 cSABPs. They are (i) metabolism of proteins (17 cSABPs), (ii) immune system (16 cSABPs), (iii) vesicle-mediated transport (16 cSABPs), (iv) signal transduction (16 cSABPs) and (v) metabolism (12 cSABPs) (Fig. 2A,B; Supplementary Table S4). Notably, five cSABPs associated with the signal transduction pathway [CAP-Gly domain containing linker protein 1 (CLIP-170), SLIT-ROBO Rho GTPase-activating protein 2 (srGAP2), transforming acidic coiled-coil-containing protein 3 (TACC3), spindle apparatus coiled-coil protein 1 (SPDL1) and diaphanous-related formin-3 (DIAPH3)], are involved in RHO GTPase-mediated activation of formin, which regulates actin polymerization^24–27^. In the immune system pathway, three cSABPs are associated with the NF-κB-mediated immune activation pathway (NEMO, p100 and HOIP). Finally, two cSABPs associated with the metabolism pathway are involved in glycolysis (ENO1 and PKM2). The full list of cSABPs associated with various pathways is presented in Supplementary Table S4.

### Interaction of ENO1 and PKM2 with salicylates

**Figure 3:**
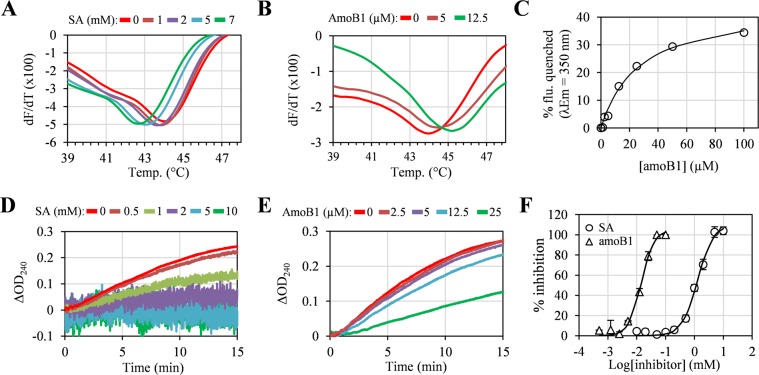
Interaction and inhibition of ENO1 with SA and amorfrutin B1 (amoB1). (**A**,**B**) Thermal stability assay of ENO1 with different concentrations of SA (**A**) or amoB1 (**B**). For the thermal stability assay, different concentrations of salicylates (SA or amoB1) were incubated with 2 µM ENO1 for 30 min at RT, then melting curves were obtained by monitoring the fluorescence at 590 nm. (**C**) Tryptophan (Trp) intrinsic fluorescence assay of ENO1 with different concentrations of amoB1. For the Trp intrinsic fluorescence assay, different concentrations of salicylates were incubated with 1 µM ENO1 for 30 min at RT, then Trp emission fluorescence was obtained (excitation/emission = 295 nm/350 nm). (**D**,**E**) Inhibition of ENO1 enzymatic activity by SA (**D**) or amoB1 (**E**). For the inhibition assays, different concentrations of salicylates were incubated with 0.1 µM ENO1 for 30 min at RT, 2.5 mM 2-PGA was added, then the production of PEP was measured by monitoring the absorbance at 240 nm. Background signals induced by reaction solutions containing salicylates and 2-PGA, but without ENO1, were subtracted to calculate ΔOD_240_. (**F**) Percent inhibition of ENO1 activity by SA and amoB1. Percent inhibition was calculated by monitoring the ΔOD_240_ at 5 min with or without salicylates. Data are mean ± SD (n = 3). Experiments were repeated three times with similar results.

For a cSABP to qualify as a true SABP, we previously specified that it should interact with SA in at least two different independent assays. Beyond crosslinking with 4AzSA in the original screen, three additional assays were used to assess the interaction between SA and the most enriched cSABP, ENO1 (Table 1). The natural salicylate amoB1 from the medicinal legume *Glycyrrhiza foetida* (commonly called licorice) also was included in these assays, as it inhibits the disease-associated activity of two previously identified SABPs, HMGB1 and GAPDH, with greater potency than SA^14,15^. Since interaction with a small molecule can alter a protein’s melting temperature (Tm), SA and amoB1 binding by these cSABPs was first assessed using a thermal stability assay^7^. A Tm of 44 °C was observed for recombinant ENO1 in the absence of SA or amoB1. As the concentration of SA increased, the Tm of ENO1 decreased; a Tm of 43.0 °C was observed in the presence of 5 mM SA (Fig. 3A; Supplementary Fig. S3A). By contrast, the Tm of maltose-binding protein (MBP), which does not bind SA^16^, was not altered in the presence of 10 mM SA (Supplementary Fig. S3B,C). AmoB1 also altered the Tm of ENO1 (Fig. 3B; Supplementary Fig. S3D). Unlike SA, however, increasing concentrations of amoB1 elevated ENO1’s Tm, with 12.5 µM amoB1 increasing the Tm to 45.1 °C.

The interaction between ENO1 and SA or amoB1 was next evaluated using the tryptophan (Trp) intrinsic fluorescence assay. This assay detects ligand-induced conformational changes in the target protein by monitoring whether the Trp-induced fluorescence of the target protein differs in the presence/absence of the ligand. Due to the high background fluorescence signal generated by millimolar concentrations of SA, the interaction between ENO1 and SA could not be assessed. In contrast, micromolar levels of amoB1 quenched ENO1’s intrinsic fluorescence without generating any background fluorescence. This enabled determination of the binding affinity for amoB1; the calculated K_d_ was 26.9 µM (Fig. 3C). Together, the thermal stability and Trp intrinsic fluorescence assays indicate that ENO1 interacts with SA and amoB1 at low millimolar and micromolar concentrations, respectively.

Binding of a molecule to a protein is required, but may not be sufficient, to modulate activity of the protein. Thus, we analyzed whether the interaction of SA or amoB1 with ENO1 altered its enzymatic activity. ENO1-mediated conversion of 2-phospho-D-glycerate (2-PGA) to PEP was inhibited by SA in a dose-dependent manner (Fig. 3D; Supplementary Fig. S4). It should be noted that as the concentration of SA increased in these assays, the absorbance pattern became wider. This phenomenon appears to be due to SA, as it was observed in samples lacking ENO1 or the substrate 2-PGA. Consistent with ENO1’s higher affinity for amoB1, this salicylate inhibited ENO1 activity at low micromolar concentrations (Fig. 3E). The calculated IC_50_ for SA and amoB1 was 1.3 mM and 15.4 µM (~84-times stronger than SA), respectively (Fig. 3F). In summary, SA and amoB1 inhibit ENO1’s catalytic activity via direct interaction.

**Figure 4:**
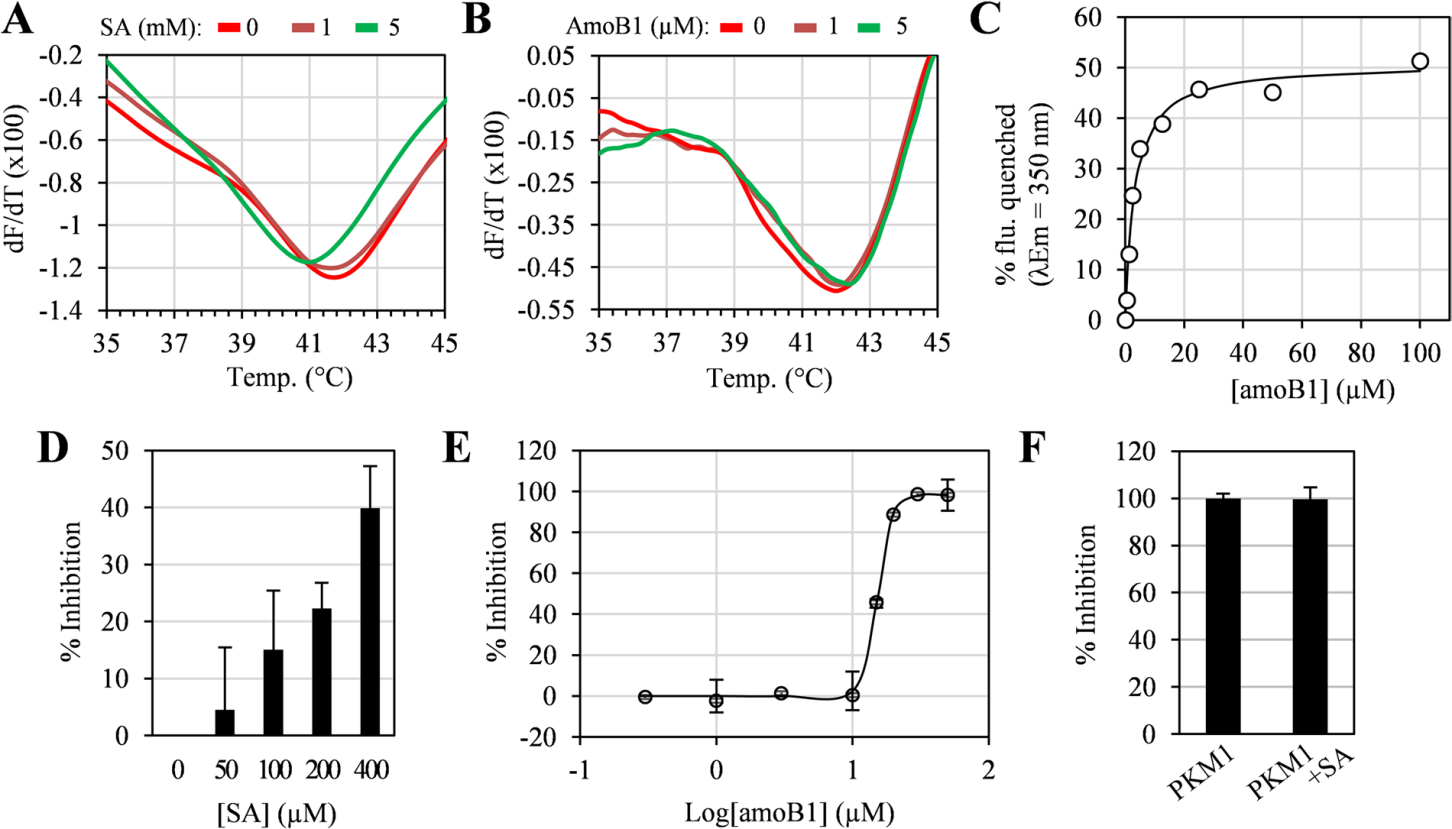
Interaction and inhibition of PKM2 with SA and amoB1. (**A**,**B**) Thermal stability assay of PKM2 with different concentrations of SA (**A**) and amoB1 (**B**). For this assay, different concentrations of SA or amoB1 were incubated with 2 µM PKM2 for 30 min at RT, then melting curves were obtained by monitoring the fluorescence at 590 nm. (**C**) Trp intrinsic fluorescence assay of PKM2 with different concentrations of amoB1. For the Trp intrinsic fluorescence assay, different concentrations of amoB1 were incubated with 1 µM PKM2 for 30 min at RT, then Trp emission fluorescence was obtained (excitation/emission = 295 nm/350 nm). (**D**,**E**) Inhibition of PKM2 activity by SA (**D**) and amoB1 (**E**). For the inhibition assays, different concentrations of salicylates were incubated with 1.3 ng/uL PKM2 for 30 min at RT, then conversion of NADH to NAD^+^ was monitored (excitation/emission = 340 nm/460 nm). Data are mean ± SD (n = 3). (**F**) Effect of SA (0.4 mM) on PKM1 enzyme activity. Experiments were repeated three times with similar results.

Thermal stability and Trp intrinsic fluorescence assays also were used to assess the interaction of SA and/or amoB1 with the second most enriched cSABP, PKM2 (Table 1). The Tm of recombinant PKM2 decreased from 42 °C to 41 °C in the presence of 5 mM SA (Fig. 4A; Supplementary Fig. S3E), while it increased from 42 °C to 42.4 °C in the presence of 5 µM amoB1 (Fig. 4B; Supplementary Fig. S3F). Due to the high background fluorescence signal generated by millimolar levels of SA, only amoB1 was tested in the Trp intrinsic fluorescence assay. AmoB1 quenched the intrinsic fluorescence of PKM2 with a calculated K_d_ of 3.2 µM (Fig. 4C). These results argue that SA and amoB1 interact with PKM2 at low millimolar and low micromolar concentrations, respectively.

To assess whether SA or amoB1 binding altered PKM2 activity, a lactate dehydrogenase (LDH)-coupled kinetic assay was employed^28^. Tetrameric PKM2 is the most active form of the enzyme, while the dimeric form is nearly inactive^29^. Thus, avoiding over-dilution of PKM2 in the assay is critical, as this prevents the dimer-tetramer equilibrium from shifting towards dimer formation. To determine an appropriate enzyme concentration, recombinant PKM2 activity was monitored in assays containing the enzyme at final concentrations of 0.3 ng/μL or 1.3 ng/μL. PKM2 activity declined rapidly after the enzyme was diluted to 0.3 ng/μL; however, it remained fairly stable at a final concentration of 1.3 ng/μL (Supplementary Fig. S5). Thus, 1.3 ng/µL PKM2 was used to test the effect of SA and amoB1 on PKM2 activity. SA inhibited PKM2 activity in a dose-dependent manner, with 400 µM SA inhibiting up to 40% of activity (Fig. 4D). Unfortunately, inhibition at higher concentrations of SA could not be tested due to the high background fluorescence signal coming from SA. AmoB1 also inhibited PKM2 activity in a dose-dependent manner. Consistent with PKM2’s higher affinity for amoB1, the calculated IC_50_ for amoB1 was 15.2 µM (Fig. 4E). Whether SA inhibits PKM2’s alternative splice variant, PKM1^30,31^, also was tested. SA did not alter the enzymatic activity of PKM1 (Fig. 4F), arguing that inhibition of ENO1 and PKM2 is not due to non-specific binding of SA.

### Effects of SA and amoB1 on the glycolytic activity of HEK293 cells

Since SA and amoB1 interact with and inhibit two glycolytic enzymes *in vitro*, we tested whether salicylates affect aerobic glycolysis *in vivo*. In normal resting cells, glycolysis converts glucose to pyruvate. Under aerobic conditions, pyruvate is funneled into the TCA cycle where it is completely oxidized and ATP is subsequently generated via oxidative phosphorylation. When oxygen levels are limiting, pyruvate is instead reduced to lactate, which allows ATP generation to continue. In general, differentiated cells only convert pyruvate to lactate under anaerobic conditions. However, activated immune cells and some highly proliferating cells, including tumor cells, exhibit high rates of lactic acid production even in aerobic conditions. This altered metabolism, known as aerobic glycolysis, is thought to play an important role in fulfilling the distinct metabolic needs of these cells^30,32^. Since HEK293 cells metabolize glucose via aerobic glycolysis, the ability of SA and amoB1 to inhibit glycolytic activity in these cells was assessed by measuring extracellular lactate levels^33,34^.

**Figure 5:**
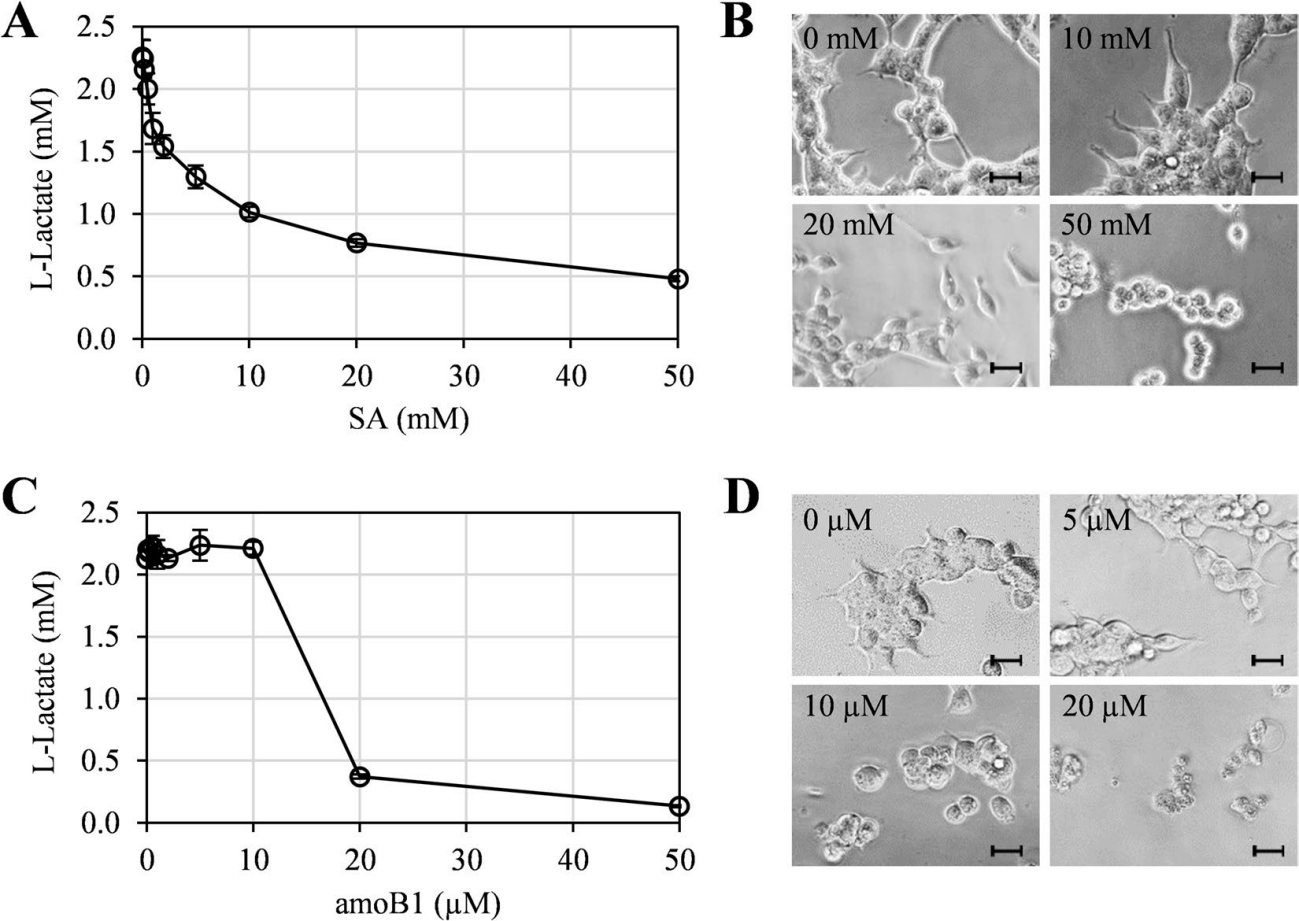
Effect of SA and amoB1 on glycolytic activity and morphology of HEK293 cells. (**A**,**C**) Suppression of extracellular lactate production by SA (0, 0.1, 0.2, 0.5, 1, 2, 5, 10 and 20 mM) (**A**) or amoB1 (0, 0.1, 0.2, 0.5, 1, 2, 5, 10 and 20 µM) (**C**). Data are mean ± SD (n = 3). (**B**,**D**) Morphology of HEK293 cells treated 15 h with the indicated concentrations of SA (**B**) or amoB1 (**D**). Scale bar = 20 μm.

After generating a standard curve to calculate the lactate concentration (Supplementary Fig. S6), glycolytic activity was monitored in HEK293 cells treated for 15 hrs with various concentrations of SA. SA treatment reduced extracellular lactate levels in a dose-dependent manner, with a 12% and 27% reduction detected at 0.5 mM and 1.0 mM SA, respectively (Fig. 5A). Notably, no detectable morphological deformation of cells was evident under the microscope with treatment up to 10 mM SA. However, cells treated with higher concentrations of SA ( ≥ 20 mM) were substantially smaller and more spherical than those treated with lower concentrations (Fig. 5B; Supplementary Fig. S7). This suggests that 0.5–10 mM SA inhibits aerobic glycolysis in HEK293 cells with little, if any, significant side effects, as assessed by cell morphology.

Analysis of glycolytic activity in the presence of amoB1 revealed that extracellular lactate levels were not substantially altered until the amoB1 concentration exceeded 10 µM. However, 20–50 µM amoB1 drastically reduced extracellular lactate levels (Fig. 5C). Cell deformation was observed at concentrations above 2 µM amoB1, with 20–50 µM amoB1 inducing cell death (Fig. 5D; Supplementary Fig. S8). Since amoB1 induced cell deformation at lower concentrations than those associated with *in vivo* inhibition of aerobic glycolysis or *in vitro* inhibition of ENO1 and PKM2, we speculate that amoB1 has additional, unknown effects that severely impact pathways and/or activities important for cell morphology and viability.

### Generation of a cSABP-disorder/disease network enables prediction of which disorders/diseases might be impacted by SA treatment

**Figure 6:**
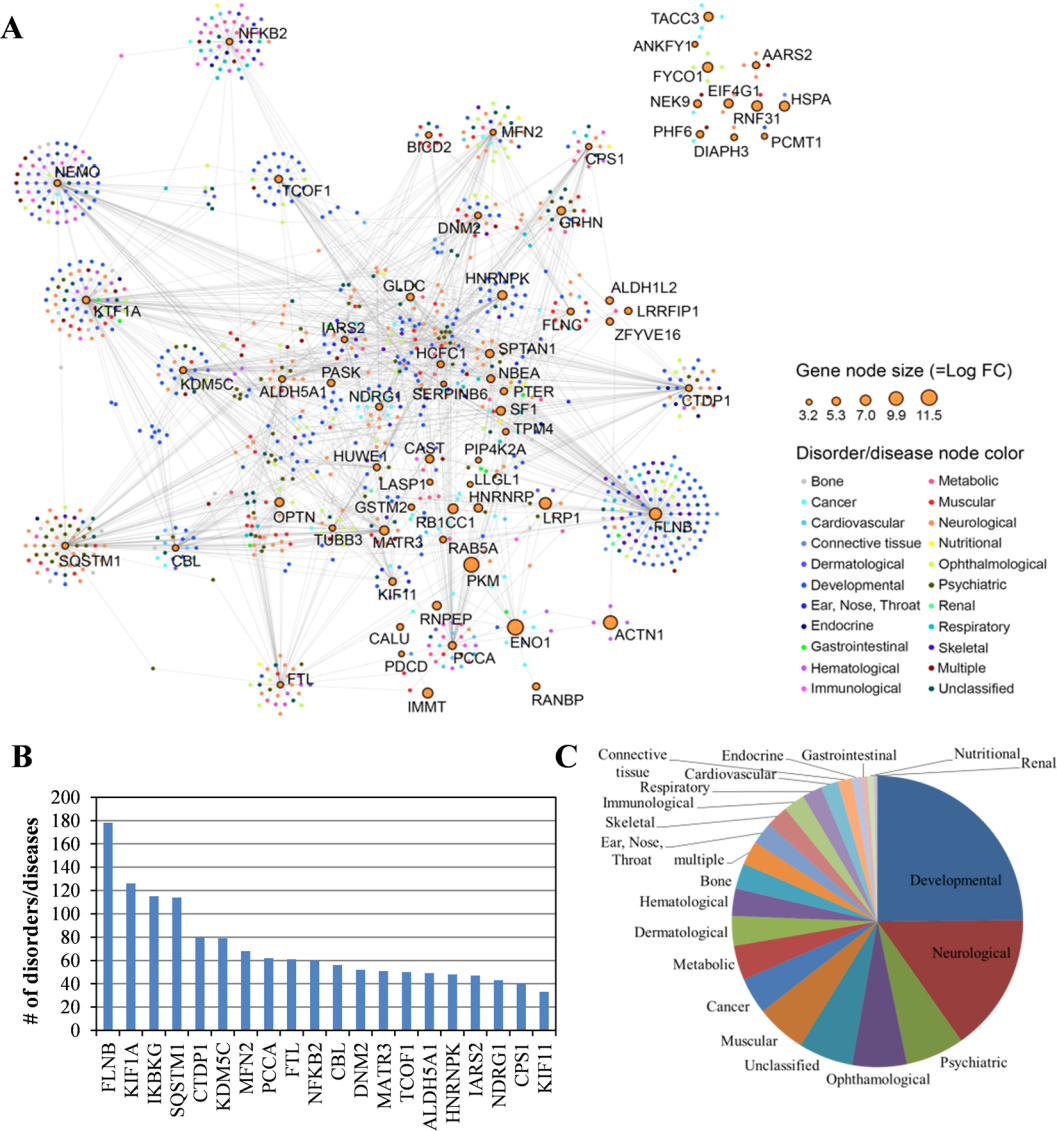
Association of cSABPs with human disorders/diseases. (**A**) Human cSABP-disorder/disease network (SDN). In SDN, each node corresponds to a distinct cSABP (orange-colored circle with black outline) or human disorder/disease (which are color-coded). The cSABP node size is proportional to the enrichment level (Log FC) of the cSABP over that in the quantitative proteome database of HEK293 cells. The different disorders/diseases were grouped into 22 primary disorder/disease classes and color coded as shown on the right. cSABP nodes are linked to disorder/disease nodes by gray lines. For a complete set of names, see Supplementary Table S5. (**B**) The number of disorders/diseases associated with the 20 most implicated cSABPs. (**C**) A pie chart showing the relative number of disorders/diseases related to the 95 cSABPs.

Several reports indicate that SA and other salicylates modulate multiple pathways through their interaction with a variety of targets, although they have only moderate to low affinity for any specific target^2,35^. To predict the systemic effects of SA on human health, the DisGeNET database v5.0, which contains a total of 696,707 gene-disorder associations as of May 2018^36^, was used to develop a list of disorders/diseases associated with the 95 cSAPBs identified by our screen. Among these, 69 cSABPs were associated with at least one disorder/disease (the gene-disorder/disease association score > 0.1), while 1,150 disorders/diseases were associated with at least one of the cSABPs (Supplementary Table S5). These disorders/diseases were assigned to 22 distinct classes based on the physiological system affected^37^. From these data, a cSABP-disorder/disease network (SDN) map consisting of two sets of nodes was generated (Fig. 6A). The gene node set corresponds to cSABPs, whereas the disorder/disease node set corresponds to disorders reportedly associated with certain cSABPs. The size of the gene (cSABP) node corresponds to the enrichment level (LogFC) of the cSABP compared to its level in the published HEK293 quantitative proteomic database. Nodes are connected to each other if they show association in the database. The 20 cSABPs associated with the highest number of disorders/diseases are shown in Fig. 6B. The full list of cSABP-related disorders/diseases is shown in Supplementary Table S5. Analysis of the SDN suggests that cSABPs are associated with multiple disorders/diseases, with the top categories including developmental (271), neurological (168), psychiatric (71), ophthalmological (66), unclassified (66), muscular (61), cancer (44), metabolic (43) and dermatological (36) (Fig. 6C).

## Discussion

To investigate how SA mediates its many pharmacological effects, we performed a genome-wide screen for SABPs using the photo-reactive SA analog 4AzSA. This ligand was employed because we and others have shown that attaching photo-reactive groups to bioactive small molecules can facilitate the screening of small molecule-binding proteins^16,17,38,39^. Through this approach, 2,604 proteins that interacted with AzSA were identified; this represents approximately 24.8% of the 10,504 proteins reported in HEK293 cells^20^. Enrichment analysis using available quantitative proteomic data from HEK293 cells identified 95 proteins that were enriched over 10-fold and therefore were more likely to be true SA targets. Indeed, SA interacted with and inhibited the activity of the two most highly enriched cSABPs, ENO1 and PKM2.

Unexpectedly, SA and amoB1 had opposite effects on the thermal stability of ENO1 and PKM2. While amoB1 binding increased the Tm of both proteins, SA binding decreased it (Figs 3A,B and 4A,B). Ligand binding generally stabilizes proteins, which increases the Tm; however, some ligands preferentially bind the unfolded state of a protein, leading to its destabilization and a reduction in Tm^40^. Compared to the relatively low molecular weight and simple structure of SA, amoB1 is a much more complex molecule with an additional alkene [(6E)-2,6-dimethyl-2,6-nonadiene], methoxy, and phenyl group on the 3^rd^, 4^th^ and 6^th^ carbons of the benzene ring of SA, respectively. These additional groups likely account for amoB1’s greater affinity for ENO1 and PKM2 as compared to SA, and the opposite effects these salicylates exert on the Tm of both proteins. Further structure-activity relationship studies are needed to understand how the additional groups on amoB1 affect ENO1 and PKM2 stability, and whether the interaction between amoB1 or SA and these proteins is mediated by the same or different binding sites.

The demonstration that SA and amoB1 bind and inhibit ENO1 and PKM2 *in vitro*, and that SA reduces the rate of aerobic glycolysis in HEK293 cells provides potentially useful insights into the anti-inflammatory and/or anti-cancer activities of salicylates. Like HEK293 cells, proliferative tumor cells and activated immune cells are heavily dependent on aerobic glycolysis to metabolize glucose^41^. The elevated production of lactate in these cells decreases the availability of pyruvate to enter the TCA cycle, which in turn reduces the rate of oxidative phosphorylation. This shift in metabolism is known as the Warburg effect, and it is a hallmark of cancer and activated inflammatory cells^32,41–44^. The function of the Warburg effect is currently unclear; however, it may benefit proliferating and activated immune cells by (i) providing a rapid, although inefficient, mechanism for generating ATP, (ii) generating biosynthetic precursors necessary to support cell growth, (iii) allowing cells to compete more effectively for limited resources, and/or (iv) promoting growth in hypoxic conditions, which can develop within tumors^32,45^.

The penultimate step in glycolysis, which involves dehydration of 2-phospho-D-glycerate to PEP, is catalyzed by the metalloenzyme enolase. In mammals, three isoforms of enolase have been identified. They are encoded by three different genes that are expressed in a tissue- and development-specific manner^46^. *ENO1* encodes the alpha isoform; it functions as a key glycolytic enzyme in many cell types. Elevated levels of *ENO1* expression have been detected in a wide variety of cancers^46^. Strikingly, knocking down *ENO1* expression impaired cancer cell growth *in vitro* and *in vivo*, and it suppressed the Warburg effect *in vitro*^47,48^. Thus, targeting ENO1 has been proposed as a novel approach for treating certain cancers^47–49^.

The final step in glycolysis is catalyzed by the rate-limiting enzyme PK, which dephosphorylates PEP to generate pyruvate and ATP^50^. Mammals contain four isoforms of PK that are encoded by two genes. The two PK muscle isozymes, M1 and M2 are generated by alternative splicing of transcripts from the *PKM* gene. Unlike PKM1, which is expressed in terminally differentiated tissues, PKM2 is the characteristic isozyme expressed in normal proliferating and embryonic cells, as well as tumor cells. Expression of the PKM2 isoform was shown to be critical for cancer cell proliferation *in vitro* and *in vivo*, as well as for the Warburg effect^30,31^. Thus, PKM2 also has been proposed as an attractive target for cancer therapy^50^.

Beyond cancer, ENO1 has been implicated in autoimmune and inflammatory diseases^46^. The recent demonstration that monocytes and macrophages isolated from rheumatoid arthritis patients express high levels of ENO1 on their surface, and that antibodies against ENO1 stimulate these cells to generate a variety of pro-inflammatory mediators, suggests one potential mechanism through which ENO1 promotes inflammation^51^. Additionally, ENO1 may regulate inflammatory responses via its function as a glycolytic enzyme. The activation of innate immune cells and the differentiation of T cells are both associated with the Warburg effect^32,41^. Given that SA and amoB1 inhibit ENO1 and PKM2 activity *in vitro* and SA suppresses aerobic glycolysis *in vivo*, our results raise the possibility that salicylates mediate their anti-cancer and anti-inflammatory effects, at least in part, by suppressing the Warburg effect.

Analyses of other highly enriched cSABPs may provide useful insights into how SA exerts its anti-thrombotic, anti-neurodegenerative, and anti-inflammatory effects. For example, SA is known to inhibit thrombin-mediated platelet aggregation, but the mechanism is not well understood^52^. Unlike aspirin, which mediates this process by inhibiting COX1 and thereby preventing generation of the pro-thrombotic eicosanoid thromboxane, SA is a very weak inhibitor of COX1 and COX2. The discovery that ACTN1 and FLNB1 are cSABPs suggests another potential mechanism. Blood clotting occurs when platelets, activated by clotting factors, reorganize their actin cytoskeleton to change shape from a smooth disc to a spiky shape that promotes adhesion to other cells^53^. Thus, if SA inhibits actin polymerization in platelets, it could have a suppressive effect on thrombogenesis. Dynamic rearrangement of the actin cytoskeleton also plays an important role in embryogenesis, cancer metastasis, wound healing, and inflammation. Further studies on SA’s interaction with cytoskeletal proteins, such as ACTN1 and FLNB1, may clarify how SA/salicylates exert beneficial effects in different diseases.

The cSABP LRP1 is predicted to play an array of physiological roles as it (i) recognizes more than 30 distinct ligands, (ii) binds a significant number of cytoplasmic proteins, and (iii) associates with and modulates the activity of other transmembrane receptors^20^. LRP1 has been implicated in several inflammation-associated diseases such as atherosclerosis, certain cancers, and neurodegenerative diseases^21,54–57^. Thus, further studies on the interaction between SA and LRP1 seem warranted, particularly in regard to LRP1’s association with neurodegenerative diseases, such as Alzheimer’s^57,58^.

Finally, HOIP is an E3 component of LUBAC, a complex that regulates NF-κB activation by binding and polyubiquitylating NEMO^22^. NF-κB activation is implicated in a wide variety of inflammatory diseases, as well as the development and progression of inflammation-induced cancer^59^. SA and aspirin are known inhibitors of the NF-κB pathway, although whether they directly modulate IKK-β’s kinase activity is unclear^10,11^. If SA is found to bind and inhibit the activity of HOIP, this would provide an alternative mechanism through which SA inhibits NF-κB activation.

By comparing the 95 cSABPs identified in our screen with the DisGeNET database v5.0, we identified 69 cSABPs associated with at least one disorder/disease and 1,150 disorders/diseases associated with at least one cSABP. Although these results require further evaluation, the SDN map generated by our results provides a valuable blueprint for future studies. For example, aspirin’s therapeutic effect on arthritis is attributed primarily to inhibition of COX-mediated prostaglandin synthesis. However, the SDN map identified five cSABPs (ENO1, FTL, IMMT, PDCD6IP and HSPA6) that were associated with various types of arthritis. Thus, the information gained from this SDN map should help (i) broaden our understanding of possible molecular mechanisms undergirding disease development, and (ii) provide an expanded roster of candidates that might be targets for SA-based drug development. In addition, the cSABP-disorder/disease network map may identify candidate proteins that are responsible for the negative side effects caused by prolonged, high dosage use of SA, including tachypnea, dyspnea, tinnitus, deafness, lethargy and seizures (Table 2)^60^.

**Table 2:** List of cSABPs associated with SA toxicity-related symptoms.

Disease	Disease IDs	Disease symptoms	cSABPs
Tachypnea	C0231835	Tachypnea	PCCA
Dyspnea	C0013404	Dyspnea	MATR3; SQSTM1; OPTN
Tinnitus	C0040264	Tinnitus	MFN2
Deafness	C0011053; C0339789; C0018772; C1384666; C3150704; C1842138; C0018777; C0155552; C0018784; C0452138; C1843156; C4021806; C4025860	Deafness; Congenital deafness; Partial hearing loss; Hearing impairment; Deafness, autosomal recessive 91; Progressive hearing loss; Conductive hearing loss; Hearing loss mixed conductive-sensorineural; Sensorineural hearing loss (disorder); Sensorineural hearing loss, bilateral; Hearing loss, progressive sensorineural; Prelingual sensorineural hearing impairment; Hearing abnormality	NDRG1; SERPINB6; FLNB; TCOF1; IARS2; IKBKG
Lethargy	C0023380; C4020875	Lethargy; Mental and motor retardation	GLDC; PCCA; CPS1; CBL; CTDP1; HNRNPK; KIF1A; TUBB3
Seizures	C0036572; C0494475; C4021759	Seizures; Tonic - chronic seizures; Generalized myoclonic seizures	CPS1; GLDC; GPHN; IKBKG; KDM5C; PCCA; SPTAN1; ALDH5A1
Confusion	C0338656; C0542476	Impaired cognition; Forgetful	CTDP1; SQSTM1

Recently, we proposed that SA plays a prominent role in the disease responses of both plants and humans via interacting with multiple targets, rather than with one or a few receptors^1,2,17,61^. In plants, over two dozen SABPs with SA binding affinities ranging from 0.045 to 15.5 mM K_d_ have been identified (http://bioinfo.bti.cornell.edu/SA2010/)^16,17^. SA levels in plants vary with the developmental stage, tissue type, and subcellular location, as well as the timing and distance from where SA synthesis was induced by an (a)biotic stress. Thus, SA likely exerts its effects on plant immunity and other physiological processes by differentially interacting with various SAPBs, depending on their affinity for SA, their location, and the local SA level^2^. Similar to these results, our genome-wide screen identified a large number of human cSABPs that, based on reactome pathway analyses, are involved in various cellular processes. In addition, different concentrations of SA in the blood are known to elicit different responses. For analgesic/antipyretic effects, the required aspirin dosage results in ~0.5 mM SA in the plasma, whereas for anti-inflammatory purposes, a dosage leading to 1~2 mM SA is appropriate; plasma concentrations of 2.5 mM SA or higher lead to SA toxicity. Recently, millimolar concentrations of SA were shown to influence the activity of two SA targets, AMPK and P300, as well as stimulate various pharmacological effects^7,12^. Taken together, these findings suggest that SA’s multiple pharmacological effects are due to the ability of millimolar concentrations of SA to interact weakly with multiple targets. These interactions are presumably influenced by (i) the affinity and location of the target protein, and (ii) the local SA concentration; interplay between these factors would lead to SA’s beneficial and/or adverse effects.

There are interesting parallels between SA and another useful natural compound, cannabidiol (CBD), which is derived from cannabis. CBD is a non-psychoactive agent that has been used to treat various diseases, including epilepsy, multiple sclerosis, inflammation, cancer, Parkinson’s disease and Alzheimer’s disease^62–69^. Unlike tetrahydrocannabinol (THC), which interacts with its cognate receptors CB1 and CB2, CBD appears to mediate its myriad effects by interacting with multiple target proteins (over 65 cellular targets have been identified)^70–72^. In summary, the results presented in this paper are the first to provide preliminary, global insights into the identities and possible functions of SABPs in human cells.

## Materials and Methods

### Screen for SABPs in HEK293 cell lysates

HEK293 cells were grown in DMEM medium supplemented with 10% heat-inactivated fetal bovine serum (FBS) at 37 °C with humidified air containing 5% CO_2_^14^. Aliquots of 5 × 10^7^ cells were collected, centrifuged, and pellets frozen at −80 °C until used. Cells were lysed for 30 min on ice with mixing every 10 min in a buffer that contained 25 mM HEPES (pH 7.4), 100 mM NaCl, 1% Triton X-100, 1 mM EDTA, 1 mM DTT, 1 mM NaVO_4_, 1 µg/ml aprotinin, 1 µg/ml leupeptin and 1X protease inhibitor cocktail (Roche). Total cell lysates were centrifuged at 20,000 × *g* for 30 min, supernatant was collected and filtered through a low protein binding polyethersulfone membrane filter (pore size 0.22 µm, Millipore), and then buffer exchanged with HNE buffer (25 mM HEPES (pH 7.4), 100 mM NaCl, and 1 mM EDTA) using a PD10 desalting column (GE healthcare). Using a slightly modified previously-described procedure^16^ the buffer-exchanged lysates were incubated with 500 µM 4AzSA (Toronto Research Chemicals) in the dark with rotation for 1 hr at 4 °C. After the incubation, the mixture was divided into two tubes; one tube was subjected to UV treatment (+UV) and the other served as the no UV control (−UV). For UV treatment, the lysate was exposed to 254 nm UV light at an energy level of 150 mJ using a GS GENE linker™ UV chamber (Bio-Rad). Unbound 4AzSA was removed via buffer exchange with a PD10 column pre-equilibrated with HNE buffer. To expose the SA moiety of 4AzSA crosslinked to the potential SABPs for accessibility to the α-SA antibody, both samples (−UV and +UV) were denatured by adding an equal volume of 8 M urea and incubated on ice for 15 min. The urea concentration was reduced to ~300 mM by dilution with HNE buffer and then proteins were concentrated using amicon ultra centrifugal filters with molecular weight cut-off of 10 kDa. The samples were then pre-cleaned with protein G agarose resin (GenScript) with rotation for 1 hr at 4 °C to remove any proteins that non-specifically bound to this resin. The supernatant recovered after centrifugation at 500 × *g* for 2 min was incubated at 4 °C overnight with rotation with protein G agarose resin coupled to α-SA antibody. The α-SA antibody-4AzSA crosslinked protein-G agarose resin complexes were washed 3 times with 1 ml of ice-cold HNE buffer containing 1/10X protease inhibitor cocktail (Roche) to remove unbound α-SA antibody. The 4AzSA crosslinked proteins were eluted from the α-SA antibody-4AzSA crosslinked protein-G agarose resin complexes using 100 μl of 10 mM SA in HNE buffer and then subjected to 12% SDS–PAGE followed by staining with MS-compatible SyproRuby staining (Thermo Fisher Scientific). The stained gel was cut into 4 pieces per sample (total 24 pieces) and then analyzed by NanoLC/MS/MS after in gel digestion with trypsin.

### In-gel trypsin digestion of SDS gel bands

In-gel trypsin digestion was performed as previously described^73^. See Supplementary Materials and Methods section for details.

### Protein identification by nano LC/MS/MS analysis

The in-gel tryptic digests were reconstituted in 20 μL of 0.5% FA for nanoLC-ESI-MS/MS analysis, which was carried out using an Orbitrap Fusion^TM^ Tribrid^TM^ (Thermo-Fisher Scientific, San Jose, CA) mass spectrometer equipped with a nanospray Flex Ion Source, and coupled with a Dionex UltiMate3000RSLCnano system (Thermo, Sunnyvale, CA)^74–76^. The gel extracted peptide samples (10 μL) were injected onto a PepMap C-18 RP nano trap column (5 µm, 100 µm i.d x 20 mm, Dionex) with nanoViper Fittings at 20 µL/min flow rate for rapid loading. The peptides were separated on a PepMap C-18 RP nano column (2 µm, 75 µm × 25 cm) at 35 °C with a 120 min gradient of 5% to 38% ACN in 0.1% FA at 300 nL/min, followed by an 8 min ramping to 90% ACN-0.1% FA and a 9 min hold at 90% ACN-0.1% FA. The column was re-equilibrated with 0.1% FA for 25 min prior to the next run. The Orbitrap Fusion was operated in positive ion mode with spray voltage set at 1.6 kV and source temperature at 275 °C. External calibration for FT, IT and quadrupole mass analyzers was performed. In data-dependent acquisition (DDA) analysis, the instrument was operated using FT mass analyzer in MS scan to select precursor ions followed by 3 second “Top Speed” data-dependent CID ion trap MS/MS scans at 1.6 m/z quadrupole isolation for precursor peptides with multiple charged ions above a threshold ion count of 10,000 and normalized collision energy of 30%. MS survey scans were conducted at a resolving power of 120,000 (fwhm at m/z 200) for the mass range of m/z 375–1575. Dynamic exclusion parameters were set at 50 s of exclusion duration with ± 10 ppm exclusion mass width. All data were acquired under Xcalibur 3.0 operation software (Thermo-Fisher Scientific).

### Data analysis

All MS and MS/MS raw spectra were used to conduct a database search against a Uniprot database for *Homo sapiens* containing ca. 20,155 entries downloaded on Oct 17, 2016 and a regular contaminant (244 entries) database using MaxQuant version 1.5.1.2. The database search was performed with an allowance for two-missed trypsin cleavage sites. The peptide mass tolerance in MS mode was set to 10 ppm and MS/MS tolerance was set to 0.6 Da. Oxidation of methionine and acetylation of protein N-terminus were specified as dynamic modifications; carbamidomethyl C was specified as a static modification. The estimated false discovery rate (FDR) thresholds for protein, peptide and modification site were specified at maximum 1%. Minimum peptide length was set at 6 and unique and razor peptide intensities were used. All the other parameters in MaxQuant were set to default values^77^.

Relative quantitation of identified proteins was determined by the Label Free Quantitation (LFQ) intensities (the output of the MaxLFQ algorithm)^78^, which were based on the (raw) intensities and normalized for combining all 4 gel slices of each sample. The LFQ intensities of each protein across all 6 samples were used for obtaining relative quantitation of proteins among 6 samples.

### Enrichment analysis

Enrichment analysis was performed to identify proteins significantly enriched by 4AzSA crosslinking and immuno-selection compared to quantitative HEK293 proteomic database^18^. See Supplementary Materials and Methods section for details.

### Purification of ENO1 and PKM2

Recombinant ENO1 and PKM2 were expressed and purified from *Escherichia coli* strain BL21 for thermal stability, Tryptophan (Trp) intrinsic fluorescence and enzyme activity assays. See Supplementary Materials and Methods section for details.

### Thermal stability assay

Thermal stability assay was performed with ENO1 and PKM2 as previously described with minor modifications^7^. See Supplementary Materials and Methods section for details.

### Tryptophan (Trp) intrinsic fluorescence assay

Binding of salicylates to ENO1 and PKM2 was assessed using a Trp intrinsic fluorescence assay as previously described^79^. See Supplementary Materials and Methods section for details.

### *In vitro* ENO1 activity assay

The catalytic activity of ENO1 was tested as previously described with minor modifications^80^. Briefly, increasing concentrations of salicylates were incubated with 0.1 µM ENO1 for 30 min at RT in reaction buffer (10 mM KCl, 5 mM MgSO_4_, 100 mM triethanolamine pH 7.4). Production of phosphoenolpyruvate (PEP) from 2-phospho-D-glycerate (2-PGA) (substrate concentration of 2.5 mM 2-PGA) by ENO1 was monitored spectrophotometrically by following the absorbance at 240 nm.

### *In vitro* PKM activity assay

PK activity was measured using a method described previously with minor modifications^28^. In brief, PK activity was measured by monitoring pyruvate-dependent conversion of NADH to NAD^+^ by lactate dehydrogenase (LDH). Increasing concentrations of salicylates were incubated with PKM2 (final concentration 1.3 ng/uL) for 30 min at RT in the incubation buffer (50 mM Tris-HCl, pH 7.5, 100 mM KCl, 5 mM MgCl_2_) before mixing with 1/10 volume of 10X pyruvate kinase reaction buffer (500 mM Tris-HCl, pH 7.5, 500 mM KCl, 50 mM MgCl_2_, 5 mM PEP (Sigma-Aldrich, P0564), 6 mM ADP (Sigma-Aldrich, A5285), 80 units LDH (Sigma-Aldrich, L1254), 10 mM DTT, and 1.80 mM NADH (SigmaAldrich, N8129)). Conversion of NADH to NAD^+^ was monitored with excitation of 340 nm and emission at 460 nm using a LS55 fluorescence spectrometer (Perkin Elmer).

### *In vivo* glycolytic activity assay

Glycolytic activity of HEK293 cells in the absence or presence of different concentrations of salicylates was measured by monitoring the level of lactate produced^33,34^. HEK293 cells were seeded in 96-well tissue culture plates at a density of  10^4^ cells/well in 120 µL of complete medium (DMEM medium supplemented with 10% heat-inactivated FBS). Plates were incubated overnight in a CO_2_ incubator at 37 °C. On the next day, cells were treated with different concentrations of SA or amoB1 in fresh complete medium. Following a 15 hr incubation in a CO_2_ incubator at 37 °C, cellular supernatants were collected and lactate levels were measured by colorimetry according to the manufacturer’s instructions for a glycolysis cell-based assay kit (Cayman, Ann Arbor, Michigan).

## Supplementary information

Supplementary Table S1

Supplementary Table S2

Supplementary Table S3

Supplementary Table S4

Supplementary Table S5

Supplementary Data
