# Supplementary material for: A genome-wide screen for human salicylic acid (SA)-binding proteins reveals targets through which SA may influence development of various diseases
Source: Sci Rep. 2019 Sep 11;9:13084. doi: 10.1038/s41598-019-49234-6 (PMC6739329; doi:10.1038/s41598-019-49234-6)
Supplement: Supplementary file 6 — Supplementary Data [file 41598_2019_49234_MOESM6_ESM.pdf]

Supplementary Material for

A genome-wide screen for human salicylic acid (SA)-binding proteins reveals targets through which SA may influence development of various diseases

Hyong Woo Choi[1,2], Lei Wang[1], Adrian F. Powell[1], Susan R. Strickler[1], Dekai Wang[1], D'Maris Amick Dempsey[1], Frank C. Schroeder[1], Daniel F. Klessig[1,*]

[1] Boyce Thompson Institute, Ithaca, New York 14853, USA.

[2] Department of Plant Medicals, Andong National University, Andong 36729, Korea.

* Corresponding author: dfk8@cornell.edu

This PDF file includes:

Supplementary Materials and Methods

References

Supplementary Figures S1 to S8

Other Supplementary Material for this manuscript includes the following:

Supplementary Tables S1 to S5 as separate Excel files

Supplementary Materials and Methods

In-gel trypsin digestion of SDS-PAGE gel bands

The protein bands from an SDS-PAGE gel (4 slices from each lane of triplicate samples from -UV and +UV treatments) were cut into ~1 mm cubes and subjected to in-gel digestion followed by extraction of the tryptic peptides as reported previously with minor modifications [1]. The excised gel pieces were washed consecutively in 200 µL distilled water, 100 mM ammonium bicarbonate (Ambic)/acetonitrile (ACN) (1:1) and ACN. The gel pieces were reduced with 70 µL of 10 mM DTT in 100 mM Ambic for 1 hr at 56 °C, alkylated with 100 µL of 55 mM iodoacetamide in 100 mM Ambic at room temperature (RT) in the dark for 60 minutes. After the wash steps as described above, the gel slices were dried and rehydrated with 50 µL trypsin in 50 mM Ambic, 10% ACN (20 ng/µL) at 37 °C for 16 hrs. The digested peptides were extracted twice with 70 µl of 50% ACN, 5% formic acid (FA) and once with 70 µl of 90% ACN, 5% FA. The two extracts from each sample were combined and lyophilized.

Enrichment analysis

For Venn diagrams depicting the presence or absence of the various proteins in each sample, iBAQ output was filtered to determine proteins that had iBAQ values of 0 and those that had non-zero values in each sample. Venn diagrams were generated separately for the three -UV and three +UV samples, using the 'VennDiagram' package in R [2]. Scaled iBAQ values for proteins present in all samples (i.e., no zero values in any -UV or +UV samples) were used to generate a heatmap using the R package 'pheatmap' (https://github.com/raivokolde/pheatmap).

For differential protein analysis, proteins in +UV samples were matched to proteins in the HEK293 dataset for comparison of iBAQ values [3]. Where multiple protein isoforms matched +UV protein IDs, data were retained for the match that contained all non-zero values. Proteins in the +UV dataset that had no match in the HEK293 dataset also were excluded from further analysis. In a few cases where multiple matches to the +UV protein contained all non-zero values, the set of three data points that had the closest average value to that of the +UV protein data was chosen, so that potential false positives would be minimized.

The curated dataset of iBAQ values for both the HEK293 [3] dataset and the +UV dataset were then used for differential expression analysis using the negative binomial model of the R library edgeR [4]. We used an FDR of 0.05. The logFC and FDR values for all proteins in the analysis were extracted using the topTags function.

Purification of ENO1 and PKM2

cDNA clones for ENO1 (clone ID: HsCD00731098) and PKM2 (clone ID: HSCD00004107) were purchased from DNASU plasmid repository (https://dnasu.org/). The coding region of each gene was amplified and cloned into pET28a vector for recombinant protein production. Recombinant ENO1 and PKM2 were produced in *Escherichia coli* strain BL21 cells as previously reported [5]. Briefly, expression of the recombinant vectors was induced with 0.1 mmol/L isopropylthiogalactoside (IPTG) for 16 h at 18°C. Cells were collected by centrifugation at 6,000 ×*g* for 30 min and stored at –20°C. For protein purification, cells were resuspended in lysis buffer consisting of buffer A (20 mmol/L Tris-HCl/8.0, 0.15 mol/L NaCl, 2 mmol/L β-mercaptoethanol (BME) and 10% glycerol) plus 0.2% NP-40, 1 mg/mL lysozyme, 1 mmol/L PMSF and 10 mmol/L imidazole. After sonication and centrifugation at

20,000 ×*g* for 1 h, soluble 6xHis-tagged proteins were purified by affinity chromatography using nickel nitrilotriacetic acid (Ni-NTA) agarose resin (Novagen). Proteins were further purified using gel filtration chromatography on a HiLoad 16/60 Superdex 200 column (GE Healthcare) equilibrated in buffer A.

Thermal stability assay

A thermal stability assay was used to examine the binding activities of ENO1 and PMK2 with salicylates as previously described with minor modifications [6]. Purified ENO1 and PKM2 were incubated with increasing concentrations of SA or amoB1 for 30 min at RT. The final concentration of each protein was 2 μM in 16 μl of reaction buffer (0.1 M HEPES, pH 7.5, 150 mM NaCl, and 5 mM BME). Four μl of a 1:200 dilution of stock SYPRO orange dye (Invitrogen) in reaction buffer was added to achieve a total reaction volume of 20 μl. Thermal melt curves were obtained by heating the protein from 20–95°C and monitoring fluorescence at 590 nm with a CFX96 Real-Time PCR Detection System (Bio-Rad). All curves were obtained in triplicate and averaged.

Tryptophan (Trp) intrinsic fluorescence assay

A Trp intrinsic fluorescence assay was used to examine the binding of salicylates to ENO1 and PMK2 [7]. Purified ENO1 and PKM2 were incubated with increasing concentrations of SA or amoB1 for 30 min at RT. The final concentration of each protein was 1 μM in phosphate-buffered saline (PBS) buffer. The mixtures were subjected to excitation wavelength of 295 nm and the Trp emission fluorescence was recorded at 350 nm using a LS55 fluorescence spectrometer (Perkin Elmer). All data were obtained in triplicate and averaged.

References

1. Yang, Y., Thannhauser, T. W., Li, L. & Zhang, S. Development of an integrated approach for evaluation of 2-D gel image analysis: impact of multiple proteins in single spots on comparative proteomics in conventional 2-D gel/MALDI workflow. *Electrophoresis* **28**, 2080–2094 (2007).

2. Chen, H. & Boutros, P. C. VennDiagram: A package for the generation of highly-customizable Venn and Euler diagrams in R. *BMC Bioinformatics* **12**, 35 (2011).

3. Geiger, T., Wehner, A., Schaab, C., Cox, J. & Mann, M. Comparative Proteomic Analysis of Eleven Common Cell Lines Reveals Ubiquitous but Varying Expression of Most Proteins. *Mol. Cell. Proteomics* **11**, M111.014050. (2012).

4. Robinson, M., Mccarthy, D. & Smyth, G. K. *edgeR Guide: differential expression analysis of digital gene expression data*. *User's Guide* (2010).

5. Choi, H. W. *et al.* Aspirin's active metabolite salicylic acid targets human high mobility group box 1 to modulate inflammatory responses. *Mol. Med.* **21**, 526–535 (2015).

6. Shirakawa, K. *et al.* Salicylate, diflunisal and their metabolites inhibit CBP/p300 and exhibit anticancer activity. *Elife* **5**, e11156 (2016).

7. Ghisaidoobe, A. B. T. & Chung, S. J. Intrinsic tryptophan fluorescence in the detection and analysis of proteins: A focus on förster resonance energy transfer techniques. *Int. J. Mol. Sci.* **15**, 22518–38 (2014).

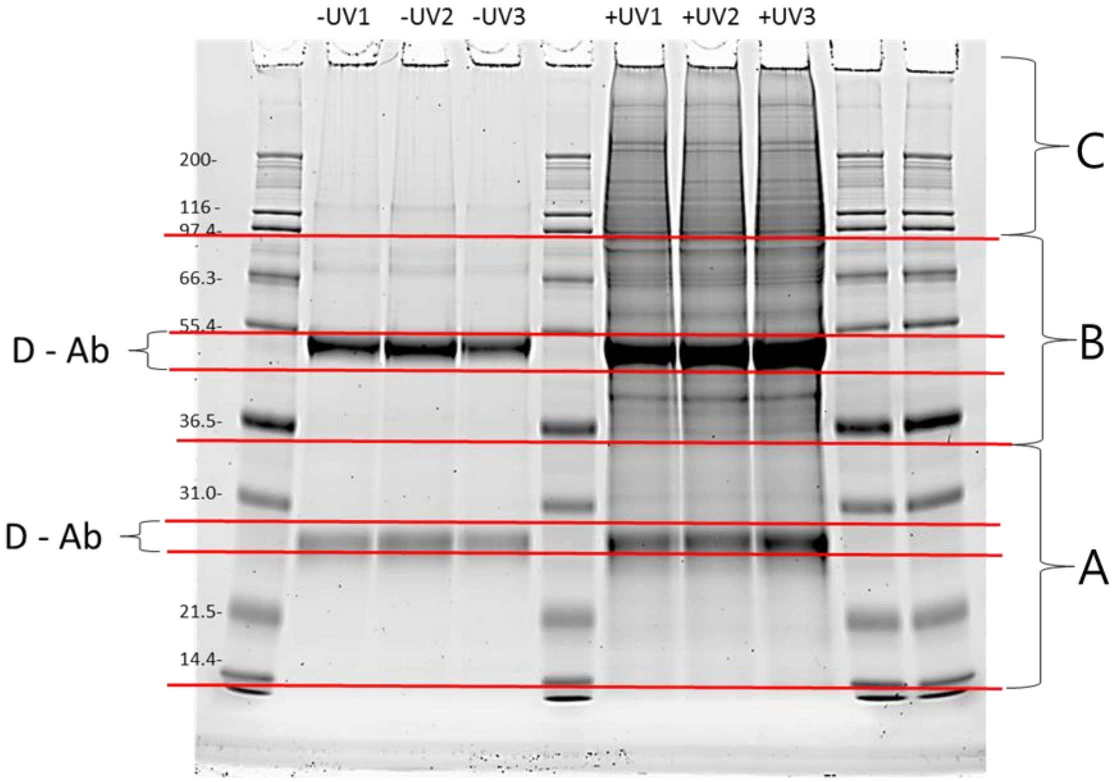

Supplementary Fig. S1. SDS-PAGE gel of proteins identified by photo-affinity crosslinking to 4AzSA, followed by immuno-selection with an anti-SA antibody. Of the three HEK293 cell lysates used for cSABP screening, half of each sample was treated with UV irradiation (+UV) and half was not (-UV). Proteins were separated on a 12 % SDS-PAGE gel. The gel was stained with SyproRuby, cut into four pieces (A, B, C, and D) per sample, and subjected to trypsin digestion followed by Nano-HPLC-MS/MS analyses.

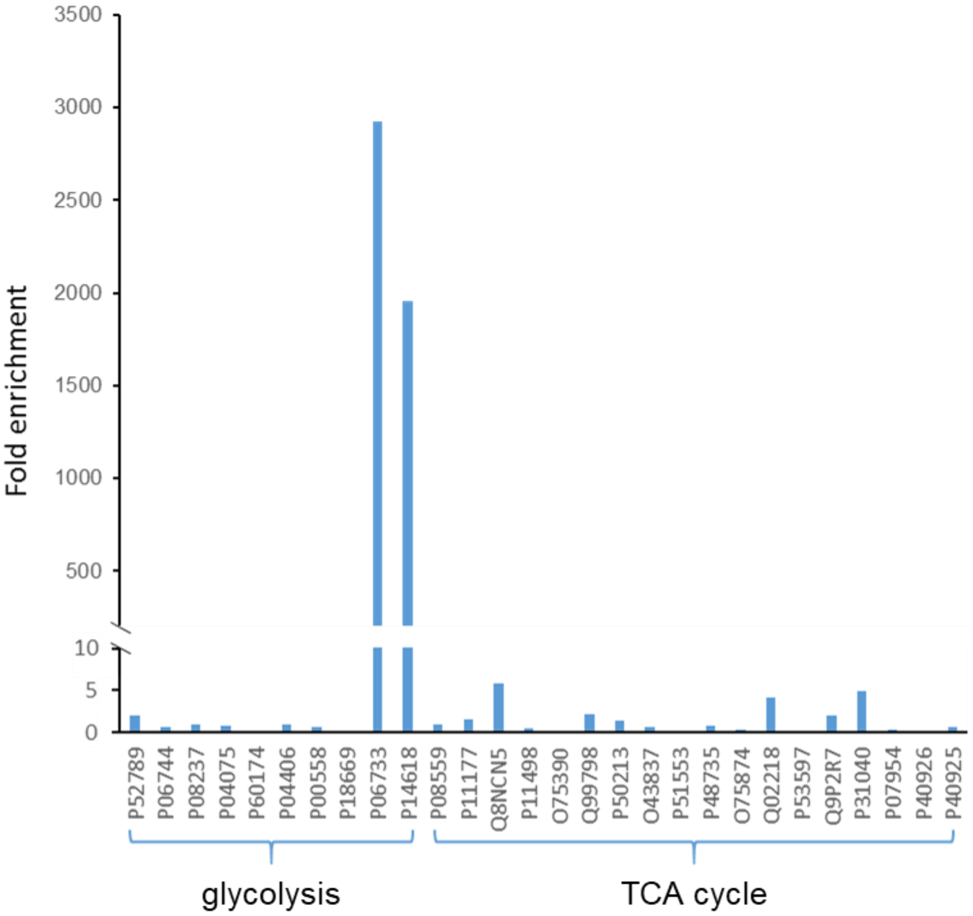

Supplementary Fig. S2. Fold enrichment of proteins which are known to participate in glycolysis and the tricarboxylic acid (TCA) cycle. Of these proteins only ENO1 (accession no. P06733) and PKM2 (accession no. P14618) were highly enriched.

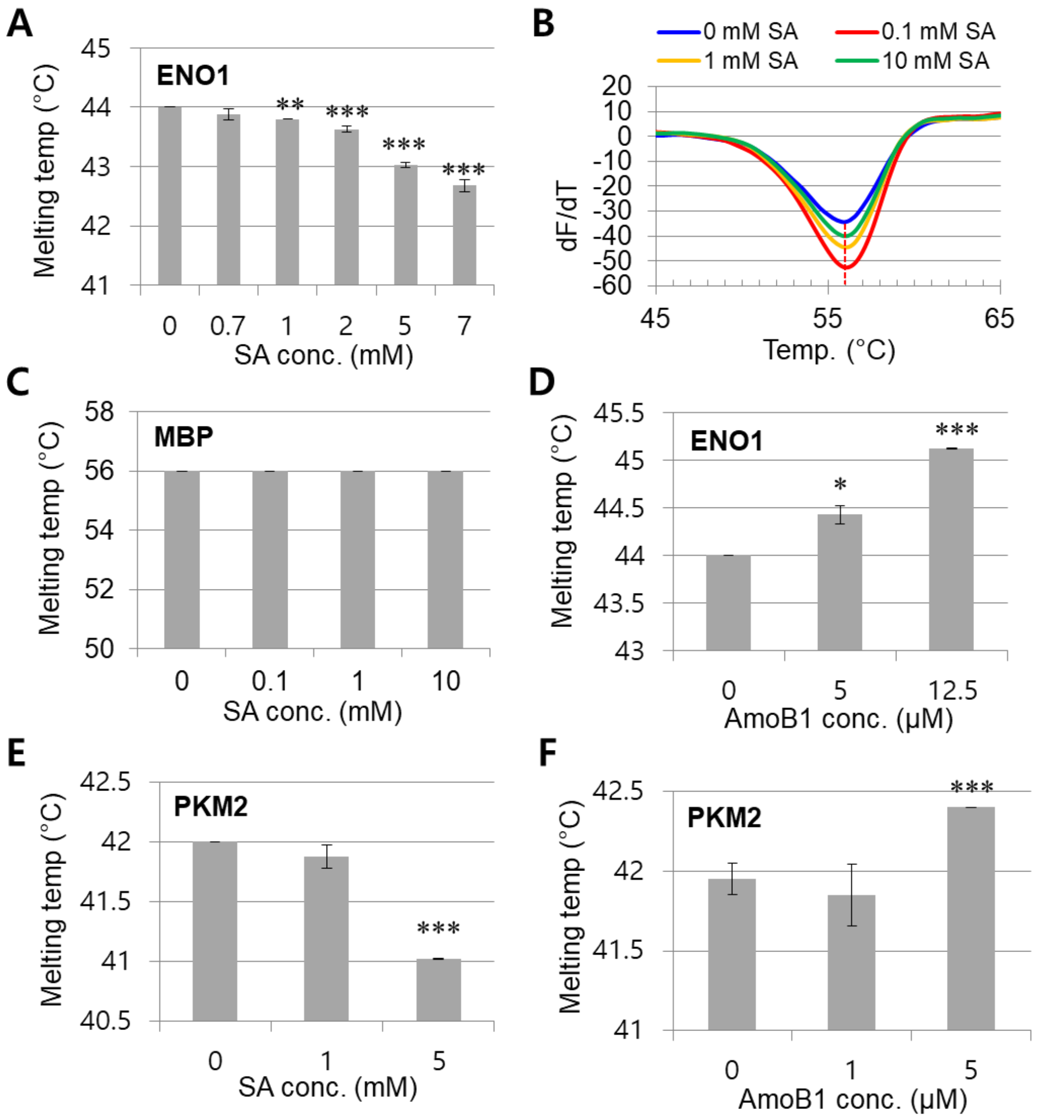

Supplementary Fig. S3. Thermal stability assay of ENO1, maltose-binding protein (MBP) and PKM2 in the presence of different concentrations of SA. **(A and E)** The melting temperature (Tm) of ENO1 **(A)** and PKM2 **(E)** in the presence of indicated concentrations of SA. **(B and C)** Thermal stability assay of MBP with different concentrations of SA. Consistent with previous analyses showing that MPB does not bind SA, the Tm of MBP was not altered in the presence of 10 mM SA. **(D and F)** Tm of ENO1 **(D)** and PKM2 **(F)** in the presence of indicated concentrations of amoB1. Experiments are repeated three times with similar results. Data are mean ± SD (n=3). * P<0.05, ** P<0.001, *** P<0.0001.

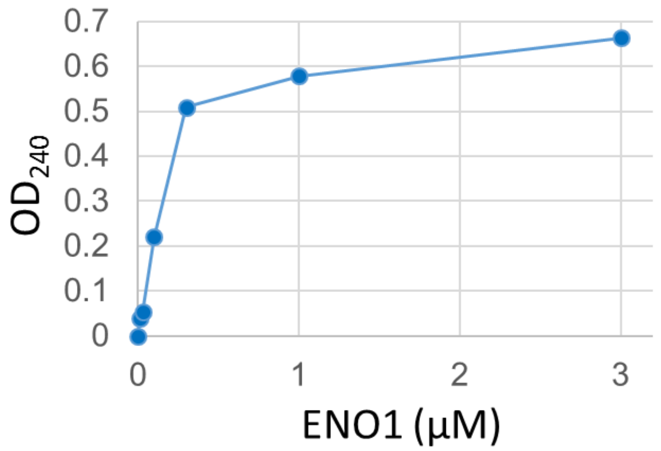

Supplementary Fig. S4. Concentration-dependent production of phosphoenolpyruvate (PEP) by purified recombinant ENO1 protein. 2.5 mM 2-phospho-D-glycerate (2-PGA) was incubated with 0, 0.01, 0.03, 0.1, 0.3, 1 and 3 μM ENO1 for 5 min and then the amount of PEP produced was determined by change in optical density at 240 nm.

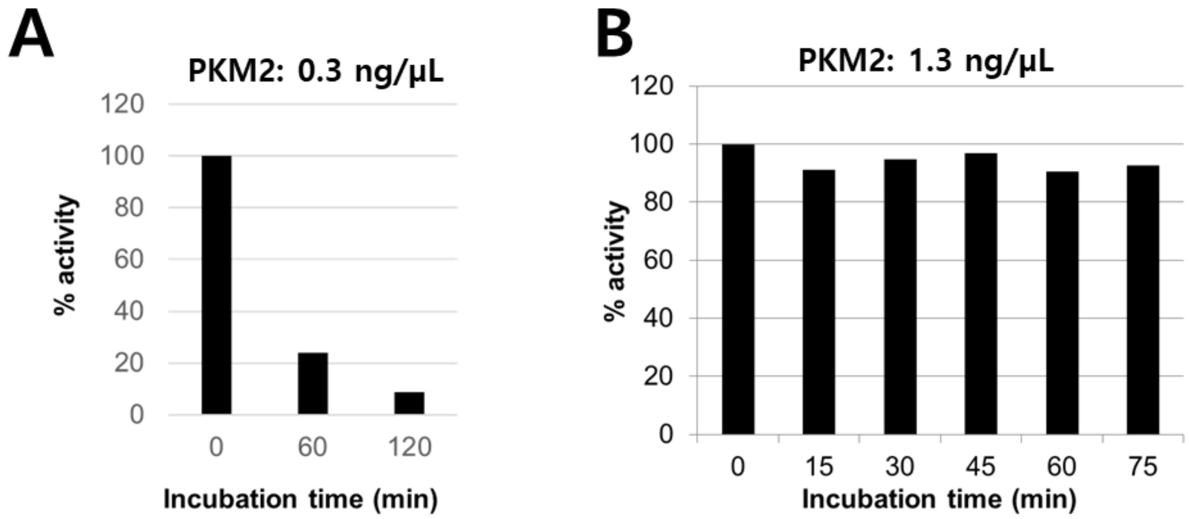

Supplementary Fig. S5. Test of pyruvate kinase activity of purified recombinant PKM2. **(A)** At a low concentration of PKM2 (0.3ng/μL) enzyme activity decreased rapidly over time. **(B)** At a high concentration of PKM2 (1.3ng/μL) enzyme activity was stable throughout the incubation time.

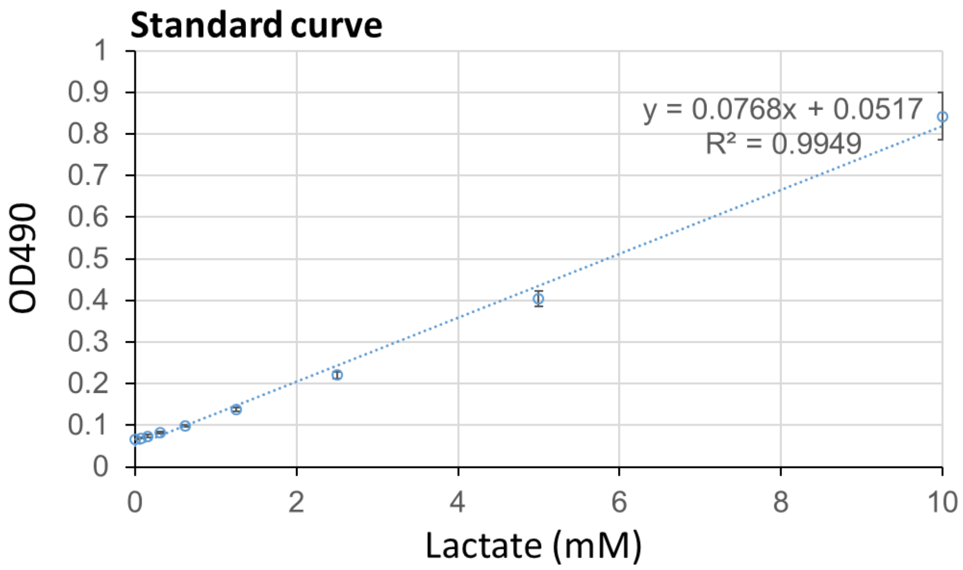

Supplementary Fig. S6. L-lactate standard curve generated for measuring the glycolytic activity of HEK293 cells.

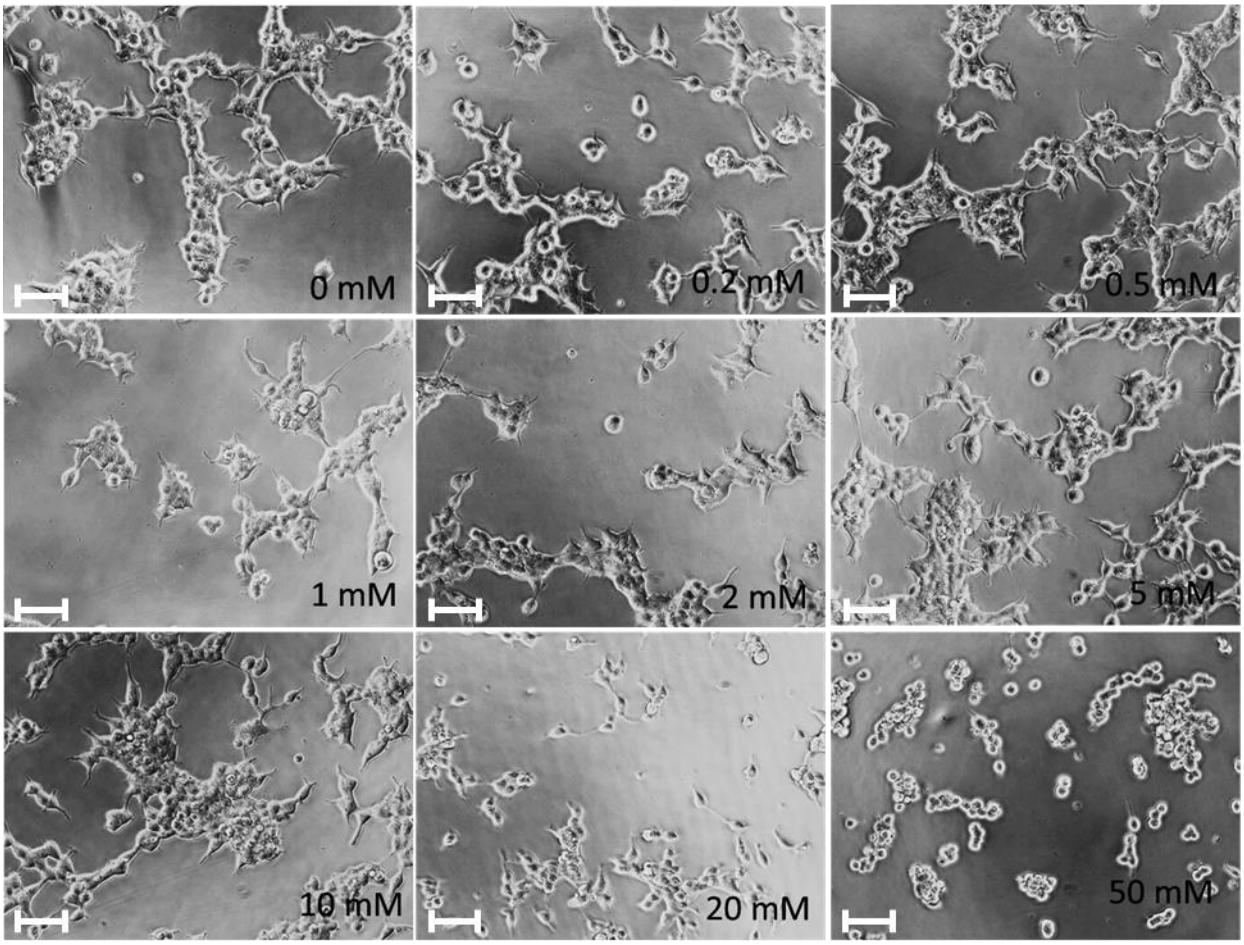

Supplementary Fig. S7. Representative cell morphologies observed after treatment with the indicated concentrations of SA. Up to 10 mM SA did not induce visible morphological changes in HEK293 cells. Scale bar = 50 μm.

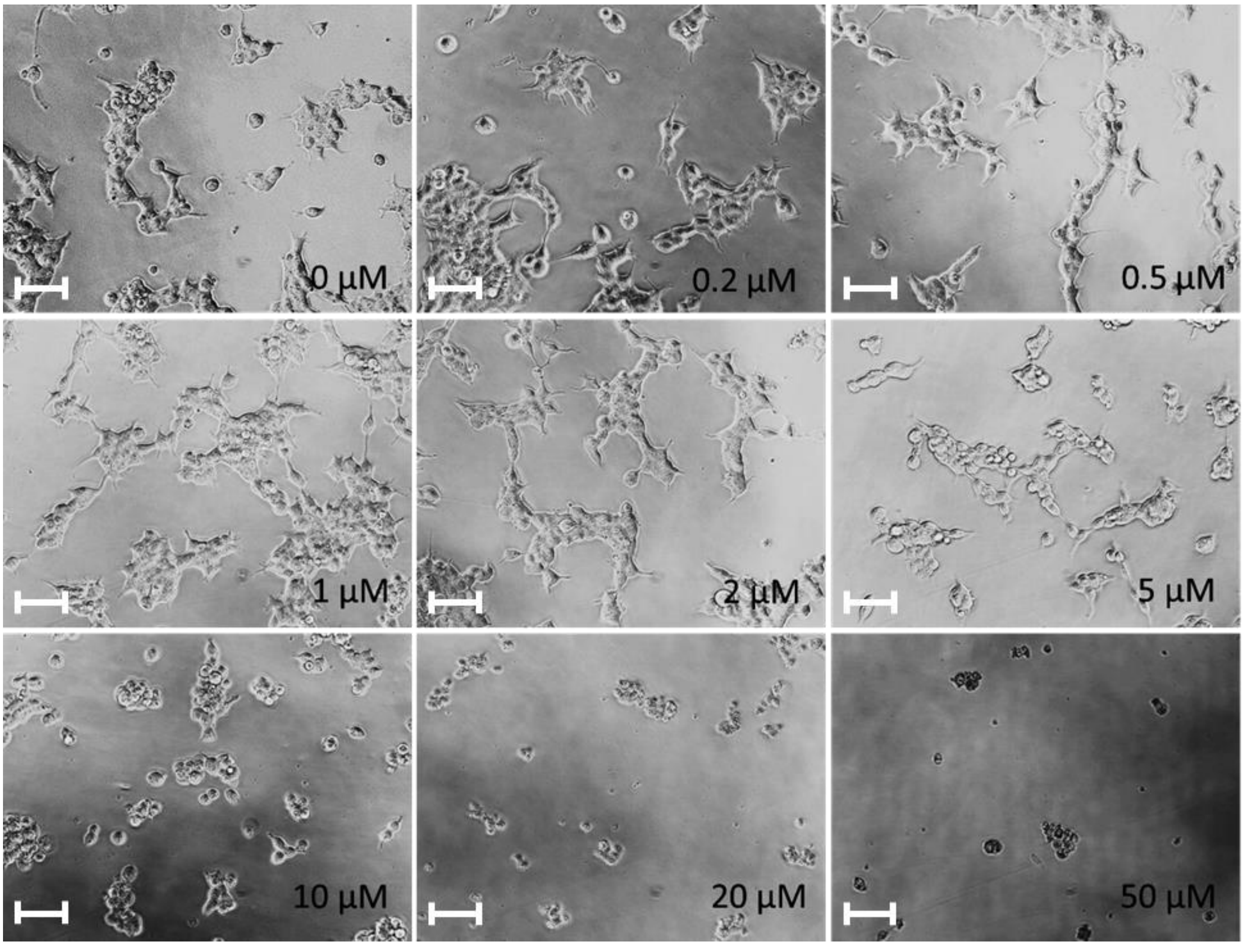

Supplementary Fig. S8. Representative cell morphologies observed after treatment with the indicated concentrations of amoB1. Up to 2 μM amoB1 did not induce visible morphological changes of HEK293 cells. Scale bar = 50 μm.
